# Estimation of amino acid contents in maize leaves based on hyperspectral imaging

**DOI:** 10.3389/fpls.2022.885794

**Published:** 2022-08-03

**Authors:** Meiyan Shu, Long Zhou, Haochong Chen, Xiqing Wang, Lei Meng, Yuntao Ma

**Affiliations:** ^1^College of Land Science and Technology, China Agricultural University, Beijing, China; ^2^College of Biological Science, China Agricultural University, Beijing, China; ^3^Department of Geography, Environment, and Tourism, Western Michigan University, Kalamazoo, MI, United States

**Keywords:** maize leaves, amino acid content, hyperspectral data, PLSR, sensitive bands

## Abstract

Estimation of the amino acid content in maize leaves is helpful for improving maize yield estimation and nitrogen use efficiency. Hyperspectral imaging can be used to obtain the physiological and biochemical parameters of maize leaves with the advantages of being rapid, non-destructive, and high throughput. This study aims to estimate the multiple amino acid contents in maize leaves using hyperspectral imaging data. Two nitrogen (N) fertilizer experiments were carried out to obtain the hyperspectral images of fresh maize leaves. The partial least squares regression (PLSR) method was used to build the estimation models of various amino acid contents by using the reflectance of all bands, sensitive band range, and sensitive bands. The models were then validated with the independent dataset. The results showed that (1) the spectral reflectance of most amino acids was more sensitive in the range of 400–717.08 nm than other bands. The estimation accuracy was better by using the reflectance of the sensitive band range than that of all bands; (2) the sensitive bands of most amino acids were in the ranges of 505.39–605 nm and 651–714 nm; and (3) among the 24 amino acids, the estimation models of the β-aminobutyric acid, ornithine, citrulline, methionine, and histidine achieved higher accuracy than those of other amino acids, with the *R*^2^, relative root mean square error (RE), and relative percent deviation (RPD) of the measured and estimated value of testing samples in the range of 0.84–0.96, 8.79%–19.77%, and 2.58–5.18, respectively. This study can provide a non-destructive and rapid diagnostic method for genetic sensitive analysis and variety improvement of maize.

## Introduction

Maize is one of the most important crops in the world (Long et al., 2017; Khanal et al., 2018; Shu et al., 2021). Nitrogen (N) is one of the most important nutrient elements in maize growth (Smil, 2002; Xu et al., 2021). The nitrogen translocation in maize leaves was mainly in the form of glutamine (Perchlik and Tegeder, 2018). The maize yield is correlated well with the amino acids in leaves, such as glutamine, glutamate, alanine, aspartate, and asparagine at the grain filling stage (Cañas et al., 2017). Therefore, accurate and rapid estimation of amino acid contents in maize leaves is of great significance in improving maize yield estimation and nitrogen use efficiency. The spectrophotometry, chemical analysis, and mass spectrometry are the main methods for determining the amino acid content. These methods can estimate a variety of amino acids and have the advantages of high sensitivity and accuracy. However, all of them need to damage samples and require complex sample processing, low throughput, and high price. The hyperspectral imaging technology provides a new method for estimating physiological and biochemical parameters of crops with the advantages of being rapid, high throughput, and non-destructive (Li et al., 2019; Mao et al., 2020). Hyperspectral imaging technology has been used for high-throughput screening of crop phenotypic traits (Zhu et al., 2020; Wang et al., 2021).

Hyperspectral imaging technology can acquire the spectral and spatial information of research objects at the same time (Zhu et al., 2019; Liu et al., 2020). Compared to digital or multispectral imaging, the advantage of hyperspectral imaging is that it can obtain hundreds of narrow bands with high spectral resolution and convenient operation. Changes in various chemical components of research objects will lead to variations in the reflectance of sensitive bands. Therefore, the spectral reflectance can quickly estimate agricultural products’ physiological and biochemical parameters (Pandey et al., 2017). The hyperspectral imaging technology has been widely applied and performed well in the non-destructive estimation of food and plant physicochemical properties (Yang et al., 2019; Huang et al., 2021), including meat, fruit, vegetation, and crop. Studies have shown that hyperspectral imaging has achieved satisfactory results in determining protein and amino acid content (Zhang et al., 2015; Egesel et al., 2016; Caporaso et al., 2018). To the best of our knowledge, little information has been conducted on applying hyperspectral imaging to molecular and biochemical parameters in plant leaves. Particularly, the research on the application of hyperspectral data in estimating the amino acid contents in fresh maize leaves is very limited.

Therefore, the study aimed to explore the feasibility of estimating various amino acid contents in fresh maize leaves using hyperspectral imaging data. Considering that the amount of nitrogen fertilizer will greatly affect the amino acid content in maize leaves, we conducted two independent experiments with variable N fertilizer applications. First, the sensitive band range and sensitive bands of each amino acid were selected by the coefficient of variation (CV) and partial least squares regression (PLSR) coefficient tests. Then, the models of 24 amino acid contents were established based on the reflectance of all bands, sensitive band range, and sensitive bands, respectively. Finally, the samples that were not involved in model construction were used to verify the model accuracy of each amino acid.

## Experimental design and data acquisition

In this study, two experiments were conducted for different N applications. The Pika-L hyperspectral imager (Resonon, United States) collected the hyperspectral images of maize leaves. The 24 amino acid contents in maize leaves were determined by liquid chromatography-mass spectrometry (LC-MS).

### Experimental design

(1) Exp1: different N application rates

Four inbred lines with great differences in nitrogen use efficiency were selected as the test varieties, including CIMBL123, CML422, 526018, and CIMBL78. The sensitivities of these varieties were as follows: CIMBL123 has a low soil and plant analyzer development (SPAD) value and yield with low nitrogen fertilizer. CML422 has a high SPAD value and yield with low nitrogen fertilizer. 526,018 has a low SPAD value and yield with high nitrogen fertilizer. CIMBL78 has a high SPAD value and yield with high nitrogen fertilizer. Maize seedlings were cultured in a complete nutrient solution with major vault protein (MVP) stone in the greenhouse until they had two outward leaves and one heart leaf. Then, three N fertilizer application rates were set up as follows: complete N treatment (N concentration was 5 mmol/L), 1/2 N treatment (N concentration was 2.5 mmol/L), and 1/4 N treatment (N concentration was 1.25 mmol/L). Before the V7 stage, 1.5 L nutrient solution was poured three times. A volume of 1 L nutrient solution was poured at the jointing stage and the male powder dispersing stage.

We collected leaf samples at the V6 stage and the filling stage. The 6th fully unfolded leaf and the leaf under the ear were cut off, and the hyperspectral images were obtained immediately. The veins and yellow areas of the leaves were then removed, and the remaining leaves were placed in tinfoil bags, frozen in liquid nitrogen, and stored in the refrigerator at –80°C for the amino acid content determinations. Six replicates were taken for the different N application experiments. A total of 144 samples were collected in Exp1.

(2) Exp2: N starvation treatment

Two inbred lines, namely, CIMBL123 and CML422, were selected as the test varieties. The Center for Crop Functional Genomics and Molecular Breeding of China Agricultural University provided all the test varieties. The maize seedlings were cultured in deionized water. In the early stage, the seedlings were cultured with a complete nutrient solution. The seedlings were treated with a low N treatment (0.05 mmol/L) when they had two leaves and one heart.

Leaf samples were collected every 3 days for a total of 13 times. The second fully expanded leaf was cut off from top to bottom, and the hyperspectral images were obtained immediately. The veins and yellow areas of the leaves were then removed, and the remaining leaves were placed in tinfoil bags, frozen in liquid nitrogen, and stored in the refrigerator at –80°C for the various amino acid content determinations. Six replicates were taken for the different N application experiments. A total of 146 samples were collected in Exp2.

### Hyperspectral images acquisition

The hyperspectral images of maize leaves were collected after each sampling. The Pika-L imaging spectrometer was used to obtain the hyperspectral images. Pika-L images provide the band range of 400–1,000 nm with a total of 300 spectral channels and 900 spatial channels. The spectral resolution was 2.1 nm. The pixel size is 5.86 μm with a field of view of 17.6°. This equipment has the advantages of low astigmatism, low distortion, and a high signal-to-noise ratio.

**FIGURE 1:**
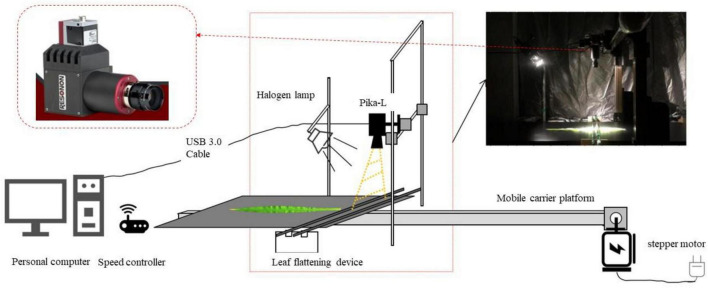
Hyperspectral images acquisition system.

A hyperspectral image acquisition system was designed and is shown in Figure 1. The system was mainly composed of Pika-L, a personal computer (PC), a halogen lamp, a mobile carrier platform, a stepper motor, a speed controller, and a blade flattening device. A halogen lamp provided stable light similar to sunlight to obtain a stable hyper-spectrum of leaves. The power of the halogen lamp is 220 W. To reduce the influence of the external environment on image quality, the hyperspectral image acquisition of maize leaves was carried out in a relatively stable dark room. Each leaf was spread flat on the platform. The hyperspectral image of the leaf was obtained directly above the leaf using the Pika-L spectrometer. Before the experiment, the hyperspectral imaging system was turned on and preheated for 30 min. The parameters of this system were set as follows: exposure time was 4.35 ms, and the speed of the electronically mobile carrier platform was 6 mm/s.

### Preprocessing of hyperspectral images

The hyperspectral images obtained include green leaves and the background. The normalized difference vegetation index (NDVI) can be used to separate green leaves from the background. NDVI is calculated by the reflectance of the near-infrared band and the red band (Formula 1) (Thenkabail et al., 2000). This study set a threshold (NDVI > 0) to distinguish the leaf pixels from the background pixels. The average hyperspectral reflectance of green leaf pixels was obtained to estimate the content of amino acids in maize leaves.


(1) N⁢D⁢V⁢I=nr⁢i⁢r-r⁢r⁢e⁢dnr⁢i⁢r+r⁢r⁢e⁢d

where *r*_*nir*_ and *r*_*red*_ are the reflectance of 780 nm and 660 nm, respectively.

### Amino acid data collection

The amino acid content was determined using LC-MS. The liquid chromatography used was ACQUITY UPLC I-Class (Waters, United States). Mass Spectrometer adopted the Q Exactive Focus system (Thermo Fisher, United States). Thermo Xcalibur 4.0 was used for data analysis. The measurement process includes the following processes: (1) Sample processing. The leaf samples were ground into powder and freeze-dried. The 20-mg freeze-dried powder was weighed as a subsample, adding 1 ml of water. Then the subsample was shaken by an ultrasonic crusher for 30 min. The subsample was centrifugally rotated for 10 min at 14,000 rpm/min.

(2) Sample derivatization. A volume of 10 μl of supernatant was taken, 50 μl of borate buffer solution and 20 μl derivative solution were added, the resultant solution was placed at room temperature for 1 min and then derived on an oscillator at 55°C for 10 min.

(3) Suction and filtration. The derived sample was cooled to room temperature and then filtered using a 1-ml syringe and filter membrane. (4) Bottling and measuring sample. The filtered sample was transferred to the glass bottle, the sample on the machine was tested, and the data were exported. (5) Drawing the standard curve of amino acids. The standard sample of amino acids was diluted to different concentrations. The peak values of molecular ions varied gradually with the increase of solution concentration, showing a linear relationship. (6) Calculating the reference value of amino acids. The Thermo Xcalibur4.0 software was used to process the mass spectrogram. The types of amino acids were determined according to the retention time and mass-charge ratio, and the peak values of molecular ions were recorded. Finally, the contents of various amino acids were obtained by putting the ion peak value into the equation of the standard curve of various amino acids.

There were 24 amino acids in maize leaves, including alanine (Ala), γ-aminobutyric acid (GABA), β-aminobutyric acid (BABA), arginine (Arg), aspartic acid (Asp), citrulline (Cit), glutamic acid (Glu), glycine (Gly), histidine (His), isoleucine (Ile), leucine (Leu), lysine (Lys), methionine (Met), ornithine (Orn), phenylalanine (Phe), proline (Pro), sarcosine (Sar), serine (Ser), threonine (Thr), tryptophan (Trp), tyrosine (Tyr), glutamine (Gln), valine (Val), and asparagine (Asn).

## Materials and methods

### Data preprocessing

Savitzky-Golay filter was used to remove noise from the hyperspectral reflectance. Savitzky-Golay filter is one of the commonly used filtering methods in spectral preprocessing and can improve the smoothness of the spectrum and reduce the noise interference (Dai et al., 2017). Due to the different magnitude of various amino acid contents, z-score standardization was used to deal with the amino acid content.

### Model construction

The estimation models of the 24 amino acid contents were constructed based on the reflectance of all bands, sensitive band range, and sensitive bands with the PLSR method. The PLSR, proposed by Herman Wold in the 1970s, cannot only reduce the dimension of the data but also solve the collinearity between the bands (Wu and He, 2014). In this study, the leave-one-out cross-validation was used to determine the number of principal components. We calculated the predicted residual error sum of squares (PRESS) of the predicted value of n–1 principal component and selected the principal components with the lowest PRESS for regression modeling. For all models, 70% (203) of the samples were used as the training set to construct the model, and the remaining 30% (87) were used as the test set to evaluate the model’s accuracy. To eliminate the random error, the modeling process was repeated 100 times, and the average result of the 100 repetitions was taken as the final result.

### Sensitive bands screening

Hyperspectral data contain hundreds of bands. Data redundancy and multicollinearity need to be addressed. Studies have shown that only using sensitive bands to establish the model can not only reduce the computational burden but also improve the accuracy and stability of the model (Wan et al., 2020). In this study, the reflectance of maize leaves was obtained at 400–1,000 nm. The greater the reflectivity variability of this band, the more sensitive it is to amino acids. The CV (Equation 2) was used to determine the sensitive band range of each amino acid.


(2) $$C\,V=\frac{\text{SD}}{\text{Mean}}\,\mathrm{x100}\%$$

where SD and mean represent the standard deviation and mean value, respectively.

Using the selected sensitive band range, we constructed the PLSR model of each amino acid and performed the regression coefficient test of the model. When screening sensitive bands, we referred to the study by Meng et al. (2013). Taking the band reflectance of the two regions as input variables, the estimation models of amino acids in maize leaves were established based on PLS regression. The regression coefficient was used to quantify the correlation between the band and the model. The larger the absolute value of the regression coefficient, the stronger the correlation between the band and the model. The absolute values of the regression coefficients of each band were sorted from small to large. The bands were removed one by one, and the model was then reconstructed. The reconstructed model was evaluated according to the PRESS. The band was counted when the PRESS value of the model was at its minimum. The above process was repeated 100 times. The bands with frequencies greater than 80 Hz used in modeling with the minimum model PRESS were taken as the sensitive bands of that amino acid.

### Model evaluation

The evaluation indices of the model include the determination coefficient (*R*^2^), root mean square error (RMSE), relative root mean square error (RE), and relative percent deviation (RPD). The average values of *R*^2^, RMSE, RE, and RPD with test set for 100 times were used to evaluate the performance and stability of models.


(3) R2=1-∑i(yi∧i-yi)2∑i(yi-i-yi)2

(4) R⁢M⁢S⁢E=1m⁢∑i=1m(yi-yi∧i)2

(5) R⁢E=1m⁢∑i=1m(yi-yi∧i)2×100%yi-i

(6) $$R\,P\,D=\frac{S\,D}{R\,M\,S\,E}$$

where m is the number of samples, y*_i_*, $\bar{y_{\text{i}}}$, $\hat{y_{\text{i}}}$ are the measured and the predicted values of various amino acid contents of sample i, and SD represents standard deviation.

## Results and analysis

### Statistics of different amino acid contents

The descriptive statistics for the entire sample are reported in Table 1. The descriptive statistics of the data included range, standard deviation (SD), and CV. The mean values of Sar, Ala, Glu, and Ser were relatively large, indicating that these amino acid contents in the samples were relatively high. The CV of Gln, Asn, Ser, and Gly was larger than the other amino acids, which may be that these amino acids were more sensitive to N treatment.

**TABLE 1:** Descriptive statistics of various amino acid contents in fresh leaves for the whole datasets (μmol/L).

Category	Max	Min	Mean	SD	CV (%)	Category	Max	Min	Mean	SD	CV (%)
BABA	64.08	0.06	7.00	6.60	94.29	Ile	66.41	0.06	8.16	7.41	90.81
Orn	11.24	0.49	5.55	4.40	79.28	GABA	51.17	0.06	10.00	8.06	80.60
Cit	16.51	0.04	4.91	3.85	78.41	Arg	19.55	2.98	7.94	3.73	46.98
Met	10.55	0.03	4.20	3.05	72.62	Tyr	40.10	2.46	9.86	5.97	60.55
His	34.40	0.39	6.49	4.56	70.26	Gln	224.48	0.09	20.93	31.58	150.88
Sar	788.29	3.75	149.96	134.36	89.60	Asn	167.89	0.03	13.11	20.83	158.89
Ala	770.64	3.75	145.37	126.77	87.21	Val	112.95	4.29	19.28	13.27	68.83
Glu	678.80	3.25	166.38	113.28	68.09	Lys	101.42	0.99	13.80	7.22	52.32
Pro	52.69	1.06	10.14	6.82	67.26	Phe	48.76	2.11	9.87	5.10	51.67
Thr	131.36	0.88	21.71	15.35	70.70	Trp	51.27	1.30	8.69	6.90	79.40
Asp	207.79	4.27	41.14	28.85	70.13	Ser	622.39	1.55	63.71	87.94	138.03
Leu	72.26	2.23	12.92	8.71	67.41	Gly	520.43	3.48	35.23	51.36	145.78

SD, standard deviation; CV, coefficient of variation.

**FIGURE 2:**
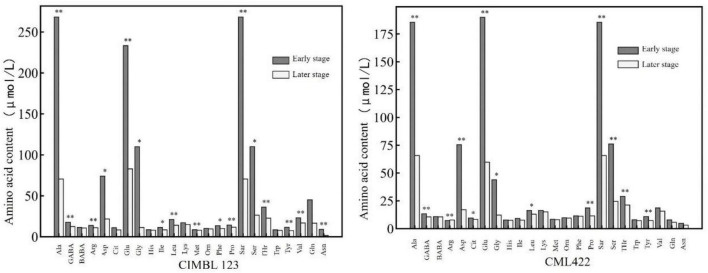
The contents of various amino acids in the leaves of two inbred lines at the early and later stages of nitrogen starvation treatment. * and ** represent significance at the 0.05 and 0.01 probability level (*p* < 0.05 and *P* < 0.01).

Figure 2 shows the comparison of various amino acid contents in maize leaves of two inbred lines sampled at the early and later stages of the nitrogen starvation experiment. In Figure 2, the early and later stages refer to the first three and the last three samples in the nitrogen starvation experiment, respectively. It can be seen that the contents of various amino acids of the two inbred lines in the later stage were lower than those in the early stage. The contents of alanine, γ-aminobutyric acid, arginine, glutamic acid, proline, sarcosine, threonine, and tyrosine in the later stage were significantly lower than those in the early stage.

### Estimation models using the reflectance of all bands

With the spectral reflectance of all bands as the independent variable and the amino acid contents as the dependent variable, we established the PLSR model of 24 amino acid contents. The validation results of the model using the test set are shown in Table 2. The estimation accuracies of β-aminobutyric acid, ornithine, citrulline, methionine, and histidine were the best, with *R*^2^, RE, and RPD of the test set in the range of 0.84–0.95, 9.68%–20.38%, and 2.52–4.95. The models of sarcosine, alanine, glutamic acid, proline, threonine, aspartic acid, and leucine had relatively good estimation accuracy, with *R*^2^, RE, and RPD of the test set in the range of 0.57–0.73, 23.23%–39.75%, and 1.53–1.95. The performance of the other amino acid models was relatively poor.

**TABLE 2:** Evaluation results of PLSR model using test set for various amino acid contents based on the reflectance of all bands.

Category	*R* ^2^	RMSE (μ mol/L)	RE (%)	RPD	Category	*R* ^2^	RMSE (μ mol/L)	RE (%)	RPD
BABA	0.95	0.80	9.68	4.95	Ile	0.54	2.56	31.50	1.48
Orn	0.94	0.77	9.87	4.64	GABA	0.50	3.90	41.56	1.43
Cit	0.92	0.96	15.95	3.74	Arg	0.49	2.48	29.34	1.42
Met	0.87	1.01	20.19	2.91	Tyr	0.49	2.61	30.44	1.42
His	0.84	1.30	20.38	2.52	Gln	0.45	13.37	71.94	1.37
Sar	0.73	53.97	39.75	1.95	Asn	0.45	11.15	90.00	1.37
Ala	0.70	50.62	39.41	1.86	Val	0.44	4.88	30.02	1.36
Glu	0.69	54.69	36.00	1.82	Lys	0.43	3.29	23.56	1.33
Pro	0.68	2.38	23.23	1.79	Phe	0.43	2.42	25.84	1.34
Thr	0.68	5.55	27.02	1.80	Trp	0.40	2.94	39.50	1.30
Asp	0.58	15.09	38.72	1.56	Ser	0.37	26.05	55.03	1.27
Leu	0.57	3.54	29.30	1.53	Gly	0.32	17.88	66.75	1.23

### Estimation models using the reflectance of the sensitive band range

**FIGURE 3:**
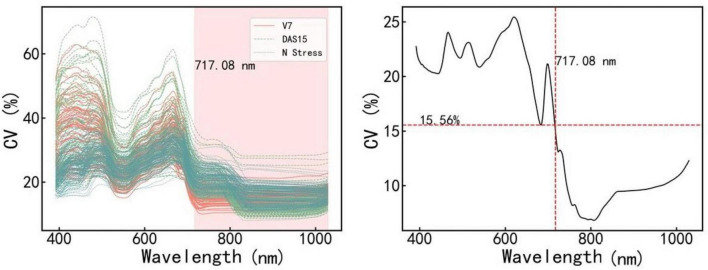
The coefficient of variation (CV) values of different samples (Left) and all samples (Right) in various bands. V7 and DAS15 represent the samples obtained at two sampling dates under different N treatments, respectively. N stress represents the samples obtained in the N starvation treatment experiment. V7 indicates that the maize is in the stage of the seventh fully unfolded leaf; DAS15 means the 15th day after maize silk.

Figure 3 shows the CV value of the spectral reflectivity of each sample (A) and all samples (B). The CV and variation range of the samples were large in the range of 400–717.08 nm and small in the range of 717.08–1,100 nm. We further constructed and validated the PLSR model of each amino acid based on the reflectance in the ranges of 400–717.08 nm and 717.08–1,100 nm, respectively. The results are shown in Table 3. The estimation model of citrulline was relatively good when using the reflectance in the range of 717.08–1,100 nm, while the estimation models of most other amino acids performed well when using the reflectance in the range of 400–717.08 nm. Therefore, the bands in the range of 400–717.008 nm were more sensitive to various amino acids than those in the range of 717.08–1,100 nm.

**TABLE 3:** Evaluation results of PLSR model using test set for various amino acid contents based on the reflectance of band ranges.

Category	400–717.08 nm				717.08–1,100 nm			
		
	*R* ^2^	RMSE (μ mol/L)	RE (%)	RPD	*R* ^2^	RMSE (μ mol/L)	RE (%)	RPD
BABA	0.96	0.75	9.19	5.16	0.96	0.76	9.23	5.13
Orn	0.94	0.73	9.22	4.91	0.95	0.67	8.48	5.48
Cit	0.92	0.95	15.69	3.82	0.93	0.92	15.75	3.90
Met	0.88	0.98	19.48	3.03	0.88	0.99	19.77	3.01
His	0.84	1.27	19.77	2.58	0.82	1.36	21.16	2.45
Sar	0.73	53.59	39.84	1.94	0.68	57.19	42.16	1.81
Ala	0.70	51.09	39.81	1.87	0.65	55.56	42.62	1.71
Glu	0.71	52.28	34.12	1.88	0.65	56.81	37.72	1.72
Pro	0.67	2.38	23.23	1.77	0.65	2.44	23.94	1.71
Thr	0.68	5.55	27.02	1.81	0.66	5.77	28.02	1.75
Asp	0.55	15.50	39.38	1.52	0.53	15.82	40.19	1.47
Leu	0.61	3.37	27.94	1.62	0.52	3.76	31.08	1.46
Ile	0.52	2.60	31.91	1.47	0.44	2.83	34.75	1.34
GABA	0.54	3.76	39.91	1.49	0.47	3.99	42.53	1.39
Arg	0.52	2.38	28.50	1.45	0.44	2.59	30.72	1.35
Tyr	0.51	2.54	29.86	1.45	0.45	2.67	31.19	1.38
Gln	0.54	12.72	67.38	1.51	0.31	15.44	84.06	1.22
Asn	0.44	10.79	91.25	1.35	0.41	1.79	96.38	1.30
Val	0.46	4.79	29.52	1.38	0.37	5.05	31.59	1.27
Lys	0.45	3.21	23.06	1.36	0.41	3.35	23.8	1.31
Phe	0.46	2.40	25.36	1.37	0.43	2.44	26.00	1.33
Trp	0.42	2.79	38.06	1.33	0.41	2.89	39.31	1.31
Ser	0.42	25.56	53.44	1.32	0.32	27.64	57.69	1.22
Gly	0.34	17.36	66.25	1.24	0.28	17.92	67.75	1.19

### Estimation models using the reflectance of sensitive bands

**FIGURE 4:**
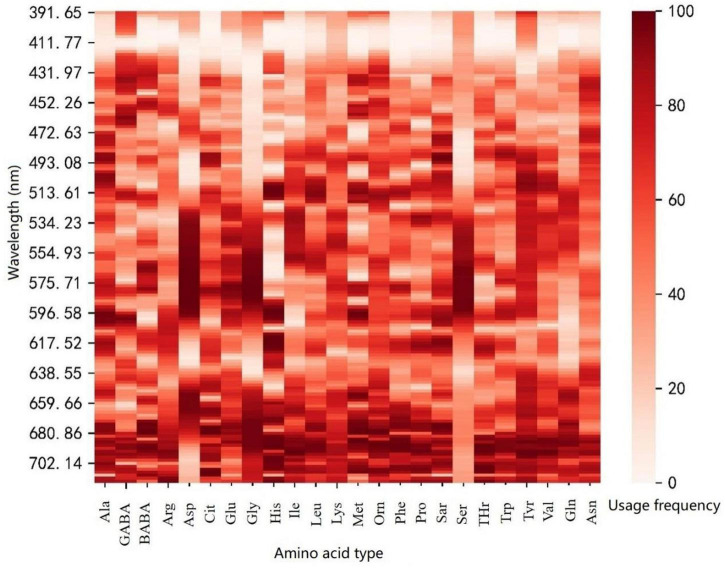
The usage frequency of each band in 100 times of modeling.

The specific sensitive bands of various amino acids were further screened in the range of 400–717.08 nm. We established the PLSR model of each amino acid using the reflectance in the range of 400–717.08 nm and performed the regression coefficient test of the model. Figure 3 shows the usage frequency of each band in 100 times modeling. The dark colors indicate the more times the band appeared and the more important the band was. As seen in Figure 4, the sensitive bands of most amino acids were mainly concentrated in the ranges of 505.39–604.95 nm and 651.21–714.10 nm.

Table 4 shows the validation results of PLSR model using test set for each amino acid based on the sensitive bands. The estimation accuracies of methionine, ornithine, sarcosine, alanine, and asparagine were improved compared with the models constructed with the reflectance in the range of 400–717.08 nm. However, the estimation accuracies of alanine, histidine, threonine, tryptophan, citrulline, β-aminobutyric acid, and aspartic acid were almost unchanged, and those of other amino acids were relatively decreased. In summary, when modeling with the reflectance of the sensitive bands, the estimation accuracies of 11 amino acids by test set were improved or equivalent to that of the model using the reflectance of band range.

**TABLE 4:** Evaluation results of PLSR model using test set for various amino acid contents based on the reflectance of sensitive bands.

Category	*R* ^2^	RMSE (μ mol/L)	RE (%)	RPD	Category	*R* ^2^	RMSE (μ mol/L)	RE (%)	RPD
BABA	0.95	0.77	9.19	4.91	Ile	0.46	2.79	34.56	1.37
Orn	0.95	0.69	8.79	5.18	GABA	0.47	3.95	42.00	1.38
Cit	0.92	0.98	16.55	3.72	Arg	0.48	2.51	29.78	1.39
Met	0.90	0.91	19.03	3.30	Tyr	0.44	2.74	32.03	1.35
His	0.84	1.30	20.20	2.52	Gln	0.38	15.13	79.25	1.28
Sar	0.73	53.05	39.69	1.94	Asn	0.46	10.99	91.06	1.36
Ala	0.70	50.53	39.69	1.85	Val	0.36	5.18	32.19	1.26
Glu	0.67	56.88	37.16	1.75	Lys	0.32	3.61	25.84	1.22
Pro	0.59	2.67	25.95	1.58	Phe	0.44	2.44	25.72	1.35
Thr	0.65	5.80	28.16	1.71	Trp	0.42	2.86	38.69	1.33
Asp	0.54	15.79	39.97	1.48	Ser	0.36	27.36	57.00	1.26
Leu	0.55	3.65	30.22	1.50	Gly	0.32	17.64	67.69	1.22

### Optimal estimation result of each amino acid content

The evaluation results of the optimal model for each amino acid and the bands used are summarized in Table 5. It generally suggests that the model estimation accuracies of β-aminobutyric acid, ornithine, citrulline, methionine, histidine, and sarcosine using test set were relatively high, with *R*^2^ more than 0.7. Among the 24 amino acids, five amino acids obtained the best estimation accuracy based on the reflectance of sensitive bands. A total of 15 amino acids obtained the best estimation accuracy based on the reflectance of the sensitive band range, of which 14 amino acids used the reflectance in the range of 400–717.08 nm.

**TABLE 5:** Summary of optimal estimate results of each amino acid content using the test set.

Category	*R* ^2^	RMSE (μ mol/L)	RE (%)	RPD	Used bands
BABA	0.96	0.75	9.19	5.16	400–717.08 nm
Orn	0.95	0.69	8.79	5.18	Sensitive band
Cit	0.93	0.92	15.75	3.90	717.08–1,100 nm
Met	0.90	0.91	19.03	3.30	Sensitive band
His	0.84	1.27	19.77	2.58	400–717.08 nm
Sar	0.73	53.05	39.69	1.94	Sensitive band
Ala	0.70	50.53	39.69	1.85	Sensitive band
Glu	0.71	52.28	34.12	1.88	400–717.08 nm
Pro	0.68	2.38	23.23	1.79	400–1,100 nm
Thr	0.68	5.55	27.02	1.80	400–1,100 nm
Asp	0.58	15.09	38.72	1.56	400–1,100 nm
Leu	0.61	3.37	27.94	1.62	400–717.08 nm
Ile	0.54	2.56	31.50	1.48	400–1,100 nm
GABA	0.54	3.76	39.91	1.49	400–717.08 nm
Arg	0.52	2.38	28.50	1.45	400–717.08 nm
Tyr	0.51	2.54	29.86	1.45	400–717.08 nm
Gln	0.54	12.72	67.38	1.51	400–717.08 nm
Asn	0.46	10.99	91.06	1.36	Sensitive band
Val	0.46	4.79	29.52	1.38	400–717.08 nm
Lys	0.45	3.21	23.06	1.36	400–717.08 nm
Phe	0.46	2.40	25.36	1.37	400–717.08 nm
Trp	0.42	2.79	38.06	1.33	400–717.08 nm
Ser	0.42	25.56	53.44	1.32	400–717.08 nm
Gly	0.34	17.36	66.25	1.24	400–717.08 nm

**FIGURE 5:**
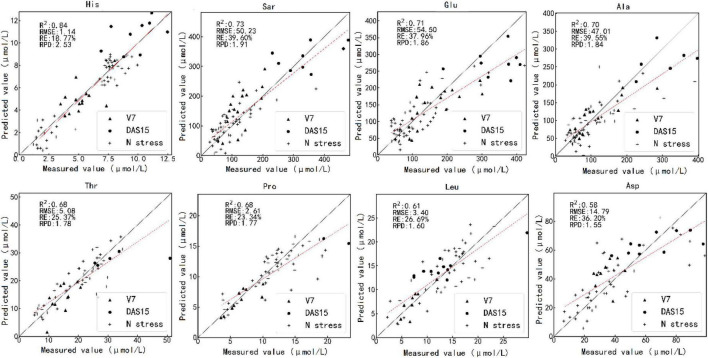
Scatterplot of the measured value against the predicted value of the various amino acid contents by the optimal estimation method using test set. The caption above each subfigure is the name of the amino acid. V7 and DAS15 represent the samples obtained at two sampling times under different N treatments. N stress represents the samples obtained in the N starvation treatment experiment. V7 indicates that the maize is in the stage of the seventh fully unfolded leaf; DAS15 means the 15th day after maize silk.

Figure 5 shows the results of testing one model randomly selected from 100 PLS regression models by the optimal estimation method. The predicted values of histidine, sarcosine, glutamic acid, and alanine were close to the measured values. The measured and predicted values of threonine, proline, leucine, and aspartic acid also matched well.

## Discussion

In recent years, spectral technology is a rapidly developed and widely used non-destructive testing technology. Amino acids can help to promote plant growth and metabolism, enhance leaf photosynthesis, and improve crop resistance to diseases and insect pests (Liu et al., 2021). The research on the application of hyperspectral data in estimating the 24 amino acid contents in fresh maize leaves is very limited. We obtained the sensitive band range and sensitive bands of each amino acid through the CV and PLSR coefficient tests, respectively. The *R*^2^ of the estimated and measured value of amino acid content was up to 0.96, among which 11 amino acids had an *R*^2^ of more than 0.6.

The physiological and biochemical traits in crop growth, such as nitrogen content, enzyme content, protein content, amino acid content, and photosynthesis rate (Sofonia et al., 2019), can reflect the growth status of the plant and be used to estimate crop yields. It is important to obtain crop physiological and biochemical phenotypes accurately, quickly, and cheaply. In terms of crop physiological phenotypes, the main indices included fresh weight, dry weight, water content, photosynthesis parameters (Vc, max, Jmax), and the internal structure of leaves (Fu et al., 2019; Gerhards et al., 2019). The main indices for crop biochemical phenotypes involved in previous studies include nitrogen content, pigment (chlorophyll a and b, carotenoid, anthocyanin), sucrose content, water content, major elements, trace elements, and protein content (Gu et al., 2018; Zhang et al., 2020). Caporaso et al. (2018) used hyperspectral imaging and PLSR to predict single kernel protein content and performed well with an *R*^2^ of 0.82. Zhang et al. (2019) combined hyperspectral imaging with PLSR, principal component regression (PCR), and support vector machine (SVM) to detect starch content in rice. The *R*^2^ of the prediction model reached 0.80. Amanah et al. (2021) used near-infrared hyperspectral imaging to realize the non-destructive detection of anthocyanin content in black rice seeds, and the *R*^2^ of the best prediction model was 0.95. These studies showed that hyperspectral technology had high feasibility in the physiological indexes of crops. We also modeled the 24 amino acid contents in maize leaves. Some of them have high accuracy and are consistent with the above research results. Similar to the above study, we also determined the sensitive bands of each amino acid through the regression coefficient test of PLSR. The difference is that before determining the sensitive band, the full spectra were divided into two regions through the CV of band reflectance, which helped reduce the redundancy of spectral information and narrow the spectral range for subsequent screening of sensitive bands for sensitive bands to increase the computation amount of model operation.

Nitrogen transfer in plants usually occurs in the form of amino acids. The proportion of amino acids produced by leaf photosynthesis varies with different amounts of nitrogen application. Crop plants mainly absorb nitrate-nitrogen (NO_3_-) and ammonium-nitrogen (NH_4_+). NH_4_+, absorbed by roots, synthesizes glutamate under the action of glutamine synthetase and then forms amino acids by glutamate synthetase and amino acid transferase. The absorbed NO_3_- forms NO_2_- under the catalysis of nitrate reductase. Most of the absorbed NO_2_- is transformed to NH_4_+ by nitrite reductase and transported to the leaf to synthesize glutamate and amino acids. The amount of nitrogen applied is closely related to the proportion of various amino acid contents in leaves. Therefore, it is feasible to use hyperspectral information to diagnose various amino acid contents in leaves.

PLSR is the most widely used traditional regression modeling method (Fu et al., 2021). Considering that the sensitive spectral band of amino acids in leaves was unclear, we first used all bands to analyze the modeling effect of various amino acids. We then reduced the spectral range by the spectral reflectance CV of all samples. It is found that the sensitivity of 400–717.08 nm reflectance to the content of various amino acid contents was much higher than that of 717.08–1,100 nm reflectance. Different N treatments led to great differences in some amino acid contents in leaves. We divided the spectrum into two regions, which helped to reduce the redundancy of spectral information and to narrow the spectral range for subsequent screening of sensitive bands. It is determined that the sensitive bands of most amino acids are mainly concentrated in the ranges of 505.39–604.95 nm and 651.21–714.10 nm. This progressive feature band screening method effectively improves the accuracy of amino acid-sensitive bands. Many studies have shown that hyperspectral information can effectively retrieve leaf nitrogen and chlorophyll content, and sensitive bands of chlorophyll content are mainly around 500 nm and 670 nm (Wang et al., 2015; Silva-Perez et al., 2018). The characteristic bands of most amino acids were mainly concentrated in the ranges of 505.39–604.95 nm and 651.21–714.10 nm, which may be mainly caused by the influence of various pigments in maize leaves, especially the chlorophyll content.

There are relatively few studies on the non-destructive detection of the amino acid content in leaves by spectral spectroscopy. N stress experiments were carried out under suitable moisture and light conditions, and our study had no water stress. However, we have only analyzed the amino acids in maize leaves. With hyperspectral imaging, it is necessary to carry out further studies to prove the feasibility of non-destructive detection of the amino acid content on the leaves of more vegetation types. The models based on the reflectance of the sensitive band range or sensitive bands performed better than those using the reflectance of all bands, showing that selecting sensitive bands helped to effectively improve the accuracy of model estimation. There are many methods to choose sensitive bands, such as successive projection algorithm (SPA) (Shorten et al., 2019), competitive adaptive reweighted sampling (CARS) (Gu et al., 2019), and instability index between classes (ISIC) (Zhang et al., 2018). Next, we will compare and analyze the similarities and differences between the bands obtained by different band screening methods and their impacts on the accuracy of the estimation model. Studies show that machine learning performs better than traditional regression in crop estimation (Chlingaryan et al., 2018; Yue et al., 2018). We will try to use a machine learning algorithm in the follow-up research further to improve the accuracy and stability of the model. The different contents of various amino acids will also lead to different responses in the narrow hyperspectral band, and the screening of sensitive bands helps estimate the content of some amino acids. This study found that imaging hyper-spectrum can estimate the amino acid contents in maize leaves, which can guide more researchers to study this topic. Of course, we are still exploring this area, and we need to test further the ability of hyperspectral technology to non-destructively estimate amino acid contents in the leaves of other crops.

## Conclusion

This study used hyperspectral imaging data to estimate the 24 amino acid contents in maize leaves. The sensitive band range and sensitive band of each amino acid were selected by the CV and PLSR coefficient tests, respectively. We found the spectral reflectance of various amino acids varied greatly in the range of 400–717.08 nm. The regression coefficient test of PLSR found that the sensitive bands of most amino acids were in the ranges of 505.39–604.95 nm and 651.21–714.10 nm. The model estimations of the 24 amino acid contents were constructed and validated based on the reflectance of all bands, sensitive band range, and sensitive bands. We selected the optimal estimation method for each amino acid. The estimation accuracy of the content of β-aminobutyric acid, ornithine, citrulline, methionine, and histidine was better than other amino acids, with *R*^2^, RE, and RPD of the test set in the range of 0.84–0.96, 8.79%–19.77%, and 2.58–5.18. The estimation accuracies of the content of sarcosine, alanine, glutamic acid, proline, threonine, leucine, and aspartic acid were normal, with *R*^2^, RE, and RPD of the test set in the range of 0.58–0.73, 23.23%–39.69%, and 1.56–1.94. The performance of the other amino acid models was relatively poor. This study can provide a reference for monitoring the traits of breeding materials based on hyperspectral technology.

## Data availability statement

The raw data supporting the conclusions of this article will be made available by the authors, without undue reservation.

## Author contributions

MS analyzed the data and wrote the manuscript. XW and YM directed the trial and provided the main idea. MS, LZ, and HC helped to collect data. XW, YM, and LM provided comments and suggestions to improve the manuscript. YM and LM edited the manuscript. All authors read and agreed to the published version of the manuscript.

## Abbreviations

N: nitrogen
PLSR: partial least squares regression
CV: coefficient of variation
MVP: major vault protein
PC: personal computer
NDVI: normalized difference vegetation index
Ala: alanine
GABA: γ -aminobutyric acid
BABA: β -aminobutyric acid
Arg: arginine
Asp: aspartic acid
Cit: citrulline
Glu: glutamic acid
Gly: glycine
His: histidine
Ile: isoleucine
Leu: leucine
Lys: lysine
Met: methionine
Orn: ornithine
Phe: phenylalanine
Pro: proline
Sar: sarcosine
Ser: serine
Thr: threonine
Trp: tryptophan
Tyr: tyrosine
Gln: glutamine
Val: valine
Asn: asparagine
PRESS: prediction error of square sum
RMSE: root mean square error
RE: relative root mean square error
RPD: relative percent deviation
SD: standard deviation
PCR: principal component regression
SVM: support vector machine
SPA: successive projection algorithm
CARS: competitive adaptive reweighted sampling
ISIC: instability index between classes.
